# How Humanistic Is Positive Psychology? Lessons in Positive Psychology From Carl Rogers' Person-Centered Approach—It's the Social Environment That Must Change

**DOI:** 10.3389/fpsyg.2021.709789

**Published:** 2021-09-28

**Authors:** Stephen Joseph

**Affiliations:** School of Education, University of Nottingham, Nottingham, United Kingdom

**Keywords:** humanistic psychology, positive psychology, person-centered approach, Carl Rogers, actualizing tendency, fully functioning person

## Abstract

Both positive psychology and the person-centered approach share a common aim to promote human flourishing. In this article I will discuss how the person-centered approach is a form of positive psychology, but positive psychology is not necessarily person-centered. I will show how the person-centered approach offers a distinctive view of human nature that leads the person-centered psychologist to understand that if people are to change, it is not the person that we must try to change but their social environment. Centrally, the paper suggests that respecting the humanistic image of the human being and, consequently, influencing people's social environment to facilitate personal growth would mean a step forward for positive psychology and would promote cross-fertilization between positive psychology and the person-centered approach instead of widening their gap.

## Introduction

It was in the late 1980's that I first became interested in what later became known as positive psychology. I was completing my doctorate research in the psychology of trauma. An unexpected finding was that many survivors reported positive changes in outlook. But there was little written in the mainstream literature about this. I wanted to find a language with which to frame my observations. Like many, I had studied humanistic psychology briefly in my undergraduate studies, but not in a way that I understood its depth and richness, so it came as a revelation to me when I discovered that the same intellectual challenges I was now grappling with, had been tackled decades ago.

Specifically, I began to see how Carl Rogers' person-centered theory of personality development could be applied to understanding how people grow following adversity. Throughout the 1990's, I studied Rogers' ideas coming to realize that what he and his colleagues had achieved from the 1950's onwards had offered a new paradigm for the psychological sciences, one that focused on how to promote human flourishing. As a result, when I first encountered positive psychology in the early 2000's, my initial reaction was to dismiss it as it seemed to offer nothing new, but I also saw the enthusiasm of my students for positive psychology, and that positive psychology was succeeding in bringing ideas about well-being back into mainstream awareness when person-centered psychology seemed to be struggling to do so. I could see that person-centered psychology was not incompatible with being interested in positive psychology, so I began to think of myself as a person-centered positive psychologist. For the past two decades I have sought to build bridges between humanistic and positive psychology, to bring the person-centered approach to my work on posttraumatic growth and authenticity, and to make the case that the person-centered approach is a form of positive psychology.

In this article I want to elaborate on what I mean when I say that the person-centered approach is a form of positive psychology. My aim is to position the person-centered approach as part of contemporary positive psychology, as well as it being part of the humanistic psychology tradition. Carl Rogers, the founder of the person-centered approach, was one of the pioneers of humanistic psychology. As such, the person-centered approach is often associated with humanistic psychology. While the relationship between humanistic and positive psychology has been contentious in the past, it is now widely accepted that positive psychology has largely followed in the footsteps of humanistic psychology. In this way, person-centered psychology can be seen as a historical antecedent to positive psychology, but what I want to show is that it is not just a branch of research, scholarship, and practice from the past; it is one that has continued and developed over the past 70 years, that now sits comfortably under the wider umbrella of positive psychology.

I would like to invite readers of this special issue to become more fully acquainted with person-centered psychology and to consider its perspective on what it means to be a positive psychologist. I will provide a brief overview of positive psychology in the context of humanistic psychology, followed by a discussion of the person-centered approach and how it offers a distinctive view of human nature, and finally, reflections on my vision for a more person-centered positive psychology. In short, the person-centered positive psychologist would look not at ways to change people but at how to change their social environment. I will show that considering the influence of the social environment as the means to facilitate personal growth would mean a step forward for positive psychology in a direction away from its individualistic and medicalized focus and would promote cross-fertilization between positive psychology and humanistic psychology. In making this argument I am reiterating and developing Linley and Joseph's (2004b) conclusion in their book *Positive Psychology in Practice* that there is a need to develop a theoretical foundation for positive psychology that offers a clear, coherent, and consistent vision of human nature, and how the agenda for the practice of positive psychology inevitably arises out of its vision. Speaking personally, my vision would be for a more person-centered positive psychology.

## Positive Psychology in the Context of Humanistic Psychology

Positive psychology was formally launched by Martin Seligman in his 1998 presidential address to the American Psychological Association (Seligman, 1999), and in the special issue of the *American Psychologist* dedicated to the topic that soon followed (Seligman and Csikszentmihalyi, 2000). Seligman later said how the idea of positive psychology came to him following a moment of epiphany when gardening with his daughter, Nikki, who was then aged five, when she instructed him not to be such a grouch. “In that moment, I acquired the mission of helping to build the scientific infrastructure of a field that would investigate what makes life worth living: positive emotion, positive character and positive institutions.” (Seligman, 2004, p. xi). But while such thinking was a refreshing change for many, these were not new ideas. The idea of focusing on the positive was an idea that was always core to humanistic psychology.

The A*merican Association for Humanistic Psychology* was founded by Abraham Maslow in 1961 (renamed the *Association for Humanistic Psychology* in 1963). Bugental (1964) put forward five basic principles of humanistic psychology, which were later adapted by Tom Greening to define the parameters of humanistic psychology: “1. Human beings, as human, supersede the sum of their parts. They cannot be reduced to components. 2. Human beings have their existence in a uniquely human context, as well as in a cosmic ecology. 3. Human beings are aware and aware of being aware —i.e., they are conscious. Human consciousness always includes an awareness of oneself in the context of other people. 4. Human beings have some choice and, with that, responsibility. 5. Human beings are intentional, aim at goals, are aware that they cause future events, and seek meaning, value, and creativity.” Humanistic psychology was known as the third force of psychology, because it recognized the limitations of its predecessors, behavioral psychology and psychoanalytical psychology. As Sutich and Vich (1969), editors of *Readings in Humanistic Psychology*, wrote:

“Two main branches of psychology – behaviorism and psychoanalysis- appear to have made great contributions to human knowledge, but neither singly nor together have they covered the almost limitless scope of human behavior, relationships, and possibilities. Perhaps their greatest limitation has been the inadequacy of their approach to positive human potentialities and the maximal realization of those potentialities” (Sutich and Vich, 1969, p. 1).

Focusing on the potentialities of being human was always a feature of humanistic psychology. For the first decade of its existence, humanistic psychology went from strength to strength (Moss, 2001). It sought to understand the nature of humanity and the problems faced in the quest to live harmoniously and peacefully together and within nature. But by the 1980's, however, the influence of humanistic psychology had begun to dwindle (Taylor and Martin, 2001). Was humanistic psychology simply ahead of its time? Had it pushed forward its more radical ideas about qualitative ways of knowing too quickly? Did becoming associated with the counterculture lose it credibility?

It seems likely that humanistic psychology lost its power and influence, not only for these reasons, but because it was “…inherently incompatible with the basic assumptions and values of contemporary mainstream psychology and with the conservative ideologies that have increasingly gained power in American culture since the 1960s” (Elkins, 2009, p. 267). By the late 1990's, humanistic psychology was largely seen as obsolete, irrelevant, and lacking in rigor by mainstream scholars (Krippner, 2001).

So it was that when positive psychology was introduced, it seemed that the ideas long championed by humanistic psychologists were now being put forward again, but it was done so in a way that was critical of humanistic psychology for what was perceived to be its anti-scientific stance, and paid scant acknowledgment to its achievements (Robbins, 2008, 2015). In 2001, in response, the *Journal of Humanistic Psychology* had a special issue containing several articles dedicated to what had become a fraught relationship between humanistic and positive psychology. Greening (2001), the then editor, opened by remarking how positive psychology had appeared as if humanistic psychology, its decades of scholarship and research, and the fact that early pioneers of humanistic psychology had themselves been presidents of the American Psychological Association, had simply not existed. Taylor (2001), in his article in the special issue, refuted Seligman's arguments that humanistic psychology was anti-scientific and that it had not generated significant research. It was also argued that positive psychology would gain from recognizing the merits of experiential, process-oriented research methodologies common to the humanistic psychotherapies (Resnick et al., 2001).

Certainly, it is clear that some of the initial comments by positive psychologists in the early days were unjustified. That said, perhaps there was also some truth in positive psychology's initial negative portrayal of humanistic psychology as it had later become. Certainly, there were aspects of the 1960's counterculture that were questionable and did no favors to humanistic psychology's standing in the eyes of mainstream psychology by becoming so closely aligned (see Grogan, 2013). As such, and as I've argued before, it was possibly a politically astute move by the positive psychologists to distance themselves from the perceived embarrassments of humanistic psychology if it was to succeed where humanistic psychology had failed in garnering mainstream attention, funding, and prestige (see Joseph and Murphy, 2013a). But as the positive psychology movement evolved, and gained footholds in the mainstream agenda, its leaders (e.g., Seligman et al., 2005) came to acknowledge, perhaps albeit reluctantly and without fully admitting their earlier critical comments were largely unfounded and misleading, that positive psychology built upon the earlier work of the pioneers of humanistic psychology (see DeRobertis and Bland, 2021). Whether intentional or not, positive psychology had helped to bring the ideas of humanistic psychology back into the mainstream.

In the early days of positive psychology, I believed that it offered the promise to bring these ideas of Rogers and other humanistic psychologists back into the mainstream agenda of scholars (see Linley and Joseph, 2004a). Almost two decades later, I think positive psychology has indeed provided an important vehicle for renewed interest in humanistic psychology. Positive psychology has become a richer and deeper form of scholarship as a result. For example, one important shift that seems to reflect the accommodation of ideas from humanistic psychology is the movement toward more eudaimonic conceptualizations of well-being as opposed to the hedonic (Joseph, 2015a). It is now not so easy to dismiss positive psychology as superficial (e.g., Ehrenreich, 2010).

One of the problems, however, in understanding what positive psychology can be, is the idea that all it offers is a corrective balance to mainstream psychology's focus on pathology. While that may be how many think of it, including perhaps how some of its pioneers originally thought of it, positive psychology has the potential to be so much more than that. As Wong (2011) wrote.

I propose that a stronger argument in support of the legitimacy of PP is that PP is much more than a corrective reaction to the perceived imbalance in the literature. Properly understood, the overarching mission of PP is to answer the fundamental questions of what makes life worth living and how to improve life for all People (p. 69).

To be more than a corrective reaction, it is essential to understand how the negative and the positive are related, and how one cannot understand the positive without the negative—what some have called positive psychology 2.0 (see Wong, 2011). There is a rich tapestry of humanistic psychology that positive psychologists are now beginning to unfold, one thread of which is the work of Carl Rogers and the person-centered approach. While Rogers is now widely recognized in positive psychology as one of the original pioneers of a more positive psychological approach, the depth and detail of his work is not in my view well-understood, and particularly how his approach offered a vision for what we now call positive psychology 2.0, or put another way, a meta-theory for positive psychology (Joseph and Linley, 2006a). In the section below I will discuss the significance for practice of Rogers' ideas—specifically how the person-centered approach proposes that if we want to change people, we need to change their social environment.

## Person-Centered Psychology

Rogers was originally a psychologist by training. In 1947 he served as the President of the American Psychological Association; the position later held by Seligman 50 years later when he founded the positive psychology movement. Throughout his life Rogers was a prolific researcher and writer, publishing numerous academic papers and books, many of which are still widely read today (see Kirschenbaum, 2007). Most known for his development of client-centered therapy (Rogers, 1951), Rogers went on to apply his theory more broadly, offering a theoretical framework encompassing personality development, psychological functioning, and helping relationships across different contexts (Rogers, 1959).

### Meta-Theory of Human Nature and Development

For the present discussion, however, the way Rogers' theory is most obviously relevant to a discussion of positive psychology is his conceptualization of the fully functioning person (1963a). Rogers (1963a) described the fully functioning person as (1) open to all their experiences, they are sensitive to the world around them, other people's reactions, and their own internal feelings, reactions, and meanings; (2) living existentially, able to be fully present in the moment; and (3) able to trust their feelings and reactions to guide them in their actions. Such a person has a non-defensive attitude, can listen to others empathically, unconditionally, communicate clearly and effectively, and respond to situations creatively. In describing the fully functioning person, Rogers provided an alternative to then dominant illness-related concepts. John Shlien, originally writing in 1956, noted:

In the past, mental health has been a ‘residual' concept – the absence of disease. We need to do more than describe improvement in terms of say ‘anxiety reduction'. We need to say what the person can do as health is achieved. As the emphasis on pathology lessons, there have been a few recent efforts toward positive conceptualizations of mental health. Notable among these are Carl Rogers' ‘fully Functioning Person' …(Shlien, 2003, p. 17).

The idea that the task should be to promote more fully functioning behavior has always been at the core of person-centered psychology (see Levitt, 2008; Joseph, 2015b). While Rogers' ideas about becoming more fully functioning may have been familiar to person-centered psychologists, they were less known to mainstream psychologists who continued to view mental health as a residual concept, until the advent of positive psychology. It should also be recognized that Rogers approached this work using the methods of traditional empirical psychological science. In this way, it is evident that person-centered psychology is a form of positive psychology.

However, and this now takes me to the main point of my article, positive psychology is not necessarily person-centered. This is because the defining feature of Rogers' person-centered theory of how fully functioning arises, is that it was grounded in a growth model (DeCarvalho, 1991; Joseph and Patterson, 2008; Joseph and Murphy, 2013b). Rogers presented a view of human nature in which becoming fully functioning was a state toward which people were intrinsically motivated. For Rogers, the person-centered approach was based on an image of the person that is basically trustworthy, and that humans are intrinsically motivated toward:

…development, differentiation, cooperative relationships; whose life tends to move from dependence to independence; whose impulses tend naturally to harmonize into a complex and changing pattern of self-regulation; whose total character is such as to tend to preserve himself and his species, and perhaps to move toward its further evolution (Rogers, 1957: p. 201).

Rogers (1959) referred to this as the actualizing tendency, a universal human motivation resulting in growth, development, and autonomy of the individual. The actualizing tendency, Rogers argued, was the one natural motivational force of human beings and which is always directed toward constructive growth (Rogers, 1963b). This will happen automatically given the optimal social environment. But too often people don't have the optimal social environment, and the unfolding of the actualizing tendency is usurped and thwarted, leading people to self-actualize in ways that are less than fully functioning. Thus, while both positive psychology and person-centered psychology might often share the same goal, how they do this may be very different and hard to reconcile (see e.g., van Zyl et al., 2016). My aim is to reflect on positive psychology from the perspective of the person-centered approach, from the point of view that the term positive psychology simply describes a broad discipline with a range of topics of scholarly and practical interest whereas person-centered psychology is a specific approach to those topics; or put another way, positive psychology is about the content, whereas the person-centered approach is about a process.

There is a famous quote from Kurt Lewin: “There is nothing so practical as a good theory” (Lewin, 1951, p. 169). I think that Rogers' (1959) approach provides a brilliant illustration of Lewin's quote. For Rogers, it followed that the logical implication of the growth model was that if people are less than fully functioning because of their social environment, then providing them with the optimal social environment would be necessary and sufficient for constructive personality change. Given the optimal social environment the person's intrinsic motivation toward fully functioning would do the rest^1^.

This ontological view of human nature is what underpins the non-directivity of the person-centered practitioner. Non-directivity is an ideological position that arises from the aforementioned fundamental assumptions of the practitioner that humans are intrinsically motivated toward personal development, differentiation, and cooperative relationships, when in optimal social environments. Non-directivity is a much-misunderstood concept. It does not mean no direction; rather it means the practitioner is not imposing their direction but trusting in and helping the client to find their own direction.

The idea that the world around us influences how we think, and feel is of course not new, and many positive psychologists have already emphasized that attention cannot only be placed on the individual. As Wong (2011) wrote.

“…you cannot live a healthy and fulfilling life in a sick world contaminated by crime, corruption, injustice, oppression, and poverty. Such evils can destroy individuals and societies like cancer cells. Positive psychology 2.0 emphasizes the need to develop good and decent people as well as a civil society by promoting meaning/virtue and overcoming and transforming negatives” (pp. 77).

Humanistic psychologists of all persuasions would no doubt wholeheartedly agree with this, but what is radically different about Rogers' person-centered approach is that it is all about the social environment—we develop “good and decent people” *through* the society we create. In short, when the optimal social environment is present, people will automatically move in directions toward fully functioning. This is the theoretical core of the person-centered approach that led to 70 years of research and scholarship concerning the operational definition of what constitutes the optimal social environment to produce constructive personality change (Cornelius-White and Motschnig-Pitrik, 2010; Murphy and Joseph, 2016). Rogers (1959) proposed that the optimal social environment was one that was experienced as unconditional, positively regarding, empathic and genuine. Taking these principles seriously in the way that Rogers' theory suggests, is an antidote to crime, corruption, injustice, oppression, and poverty. Imagine if that was the attitudinal climate experienced by children in their homes and schools, and by adults in their workplaces and in all other areas of their life.

### Applications: Prevention, and Cure

Up to this point, I've deliberately avoided the topic of psychotherapy as I wanted to make it clear that Rogers' theory is about the social environment and its influence on a person's psychological development, and in this way show how applications of his theory may be upstream in education, parenting, public policy, and so on, concerned with prevention, and not just downstream in psychotherapy and clinical psychology, dealing with problems after they arise. Applications of Rogers' (1959) theory are as much about ensuring that the unfolding of the person's actualizing tendency is not usurped and thwarted in the first place, as it is about the self-righting process subsequently.

Also, because psychotherapy and clinical psychology, more generally, are often understood from the perspective of an illness ideology, as involving activities in which one person (the therapist) attempts to change another in some predetermined way (the client), discussion of Rogers' (1959) theory in the context of therapy can be misunderstood, as if the necessary and sufficient conditions are something that one person does to another to get them to change. In this way, I wanted to situate the discussion outside the therapeutic context to avoid this misunderstanding. As such, it might therefore surprise some readers to think of Rogers' conditions as describing a social environment as more often his theory is caricatured as something the therapist does to a patient.

Regardless of the nature of the application of Rogers' approach, however, it is not about doing something to people. The attitudinal conditions of unconditionality, positive regard, empathy and genuineness come together to create the fundamental non-directive attitude of the practitioner, which because of his or her trust in the agency of the client, means that they do not intervene, and have no intention of intervening. As Bozarth (1998) wrote of client-centered therapy:

“The therapist goes with the client, goes at the client's pace, goes with the client in his/her own ways of thinking, of experiencing, or processing. The therapist cannot be up to other things, have other intentions without violating the essence of person-centered therapy. To be up to other things – whatever they might be – is a ‘yes, but' reaction to the essence of the approach. It must mean that when the therapist has intentions of treatment plans, of treatment goals, of interventive strategies to get the client somewhere or for the client to do a certain thing, the therapist violates the essence of person-centered therapy (Bozarth, 1998, pp. 11–12).

In creating an empathic, unconditional, and congruent social environment, the therapist is not trying to change the person, has no agenda for the person whatsoever, but they trust that given the optimal environment the person will change toward becoming more fully functioning. Unlike other psychologically based interventions, the person-centered practitioner is not doing anything to the person, they have no agenda for the person to change in any particular way, rather the practitioner's only agenda is for themselves to be able to create a social environment characterized by these conditions. This remains a revolutionary idea in psychology that remains underappreciated in my view, perhaps because while Rogers' theory of therapy is well-known, it is less well-understood that it is about changing the social environment, not the person. And this is what makes it a radically different form of practice to most other psychological interventions, which focus on changing the person. This can be difficult to understand if looking at the person-centered approach from outside its paradigmatic stance. But imagine if you truly believed that people would only move in directions toward becoming more fully functioning when they experience themselves in the optimal social environment.

Originally writing about therapy, Rogers (1959) soon developed his thinking more widely into how the same optimal social environment could be facilitative of growth in a range of contexts. Psychotherapy and clinical psychology are obvious applications of the person-centered approach, but in these contexts, it is about a self-righting process, helpful to people whose tendency toward actualization has already been usurped and thwarted. Of more importance, in my view, is that positive psychology expends its energy on upstream interventions, to facilitate people's psychological development in the first place, such that the eventual need for psychotherapy and clinical psychology is reduced. In this respect, education is the most powerful institution in the world for shaping the future of humanity because of its influence on how each generation comes to view what matters, and what to prioritize and to value.

Recent years have seen much interest in positive psychology applications to education and the development of the new subfield of “positive education” (Seligman et al., 2009). Positive education is a relatively new initiative, but its aims are similar to those of person-centered education, as developed by Carl Rogers in his subsequent writings about the applications of the person-centered approach. In 1969, Rogers published his influential book *Freedom to Learn* (Rogers, 1969), in which, building on his earlier writings, he set out his full philosophy of education: in essence, that human beings have a natural urge to learn, that this most readily happens when the subject matter is perceived as relevant to the student, that learning involves change and as such is threatening and resisted; that learning is best achieved by doing, and that the most lasting learning takes place in an atmosphere of freedom in which students were trusted to be autonomous learners. In essence, the goal of education should be to assist people to learn to be self-determining; to take self-initiated action and to be responsible for those actions; to be able to adapt flexibly and intelligently to new problem situations; internalize an adaptive mode of approach to problems, utilizing all pertinent experience freely and creatively; cooperate effectively with others in these various activities; and work, not for the approval of others, but in terms of their own socialized purposes. To adopt other goals in which the teacher has a pre-determined intention that the student should change in any particular direction was seen from Rogers' person-centered perspective as contradictory to the act of nurturing self-determination.

While Rogers' influence has been greatest in the field of psychotherapy, it is I would argue his contributions to education which are the most significant and important for the modern world. However, Rogers' writings on person-centered education have received little attention in the positive education literature. Positive education, whilst offering a new focus on human flourishing, does not challenge traditional education with its largely teacher-centered approach. Rogers' view on education was that it was this teacher-centered approach that was itself the problem that thwarted and usurped developmental processes and stifled creativity and curiosity. Both person-centered education and positive education have a shared focus on human flourishing. But what makes person-centered education different to positive education is its clear ontological stance that people are their own best experts, and the resultant hypothesis that with the right social environment, students will be self-determining and move in autonomous and socially constructive directions (see Joseph et al., 2020).

All the different applications of the person-centered approach—whether downstream in the domains of clinical psychology, coaching, counseling, conflict resolution, psychotherapy; or upstream in business, education, encounter groups, leadership, management, parenting, or policy, are all about changing the social environment, because they are grounded in a vision of humanity in which people are always striving toward becoming fully functioning, a tendency which will automatically be released when the social environment is optimal. This is what makes the person-centered approach distinctive, the fact that its interventions are always about changing the social environment and not about changing the person. And in changing the social environment, people will change in a way that is toward becoming more fully functioning. In turn, more fully functioning people, by definition, will create more facilitative social environments for others (Motschnig-Pitrik and Barrett-Lennard, 2010). This way of thinking is what I believe would make for a more person-centered positive psychology. But, as already indicated, a more person-centered positive psychology involves more than a simple change of focus from the individual to the social, it also challenges us to think from a different paradigmatic stance and to ask questions about the positionality of positive psychology, its politics, and its subtle use of power over others.

## Toward a More Person-Centered Positive Psychology

In this final section I will offer some reflections on positive psychology from the perspective of person-centered psychology. In so doing, I hope to show how a consideration of the person-centered approach leads to questions about positionality, politics, and power in positive psychology.

### The Unspoken Positionality of Positive Psychology

One criticism that I have heard leveled against Rogers' theory many times is that it is an ideological position. This argument implies however that there is a neutral position that one could take while waiting for that evidence. But as Burr (2015, p. 172) wrote: “No human can step outside their humanity and view the world from no position at all, and this is just as true of scientists as of everyone else.” All interventions in psychology represent ideological positions and this is one of the lessons to be learned for positive psychology as it moves forward. All forms of psychological practice and policy are grounded in a vision of the human being (Joseph, 2017). But for positive psychology, if not the growth model, what model?

To illustrate what I mean, first, all constructs used in research are derived from theories that represent an ideological position, whether expressed implicitly or explicitly, and in turn, the choice of which constructs to investigate represents one's own ideological views. Second, how we interpret the implications for practice from research is ideological. For example, research shows that greater authenticity leads to greater well-being. The implication is that increasing levels of authenticity would be desirable. But what sort of intervention should be designed to promote authenticity? There is nothing inherent in the research finding itself that presupposes the nature of the intervention, whether it be through changing the social environment or by altering the person's thoughts, feelings or behaviors. If one holds to the growth model, then changing the social environment will make sense. But if it is thought that people need instruction from others, then introducing interventions targeted directly at somehow pushing the person toward authenticity will make sense.

In this way, the choice of intervention only looks like it arises out of the research findings if one thinks of oneself as taking a neutral position. Interventions are always ideologically driven and based on the researcher's or practitioner's assumptions about human nature. And those assumptions about human nature are baked into the design of research and the language used to discuss findings. Of course, subsequent research can show support or fail to show support for an intervention, and in that way, we can be guided subsequently by research. If changing the environment doesn't work, then maybe we need to push. But, if there is no neutral position, which ideological position should be our default setting? Furthermore, out positionality also determines what factors are deemed appropriate as targets for intervention. In sum, most positive psychology interventions involve directive interventions targeted at changing the person in ways decided upon by the practitioner as best for the client, which presupposes an ideological position that runs counter to a growth model.

### The Hidden Politics of Positive Psychology

If we reflect on one reason for the demise of humanistic psychology being its clash with conservative ideologies (Elkins, 2009), I believe we also learn about the success of positive psychology. One of the features of the conservative ideology is its focus on individualism, and it is a focus on individualism that has led to the rise of a culture in which positive psychology research has been used to promote mindfulness in school children, to deal with the stressors of failing educational systems, resilience training in workers to help them cope with punitive workloads, and well-being applications to help people manage the stresses of economic insecurity (Joseph, 2020). The person-centered psychologist would see the challenges in such situations to be how to create more growth promoting climates in schools, and workplaces, and in everyday life, how to build more empathic, genuine, and unconditional relationships in which people can be autonomous and free from coercion and control, and thus able to express themselves in a more socially constructive way. This too could be the research agenda for positive psychology if it took seriously a model of growth as its paradigm.

The word paradigm is often overused to refer to new ideas and practices, but its real meaning is that of a world view underlying the theories and methodology of a particular scientific subject. Within the history of psychology, the growth model of person-centered psychology represented a genuine paradigm shift from the first and second forces in psychology, the behaviorist image of the human being as a blank slate on which anything could be written or the psychoanalytical view of the human being as driven by destructive impulses (see DeCarvalho, 1991). I believe that positive psychology was a welcome shift in the everyday business of mainstream psychology, but as Seligman (2004) made clear, it was not a paradigm shift. It continued to operate within the same world view as mainstream psychology. Thus, despite the language of positivity, it appears to me that positive psychology as a movement, largely continues to operate within models that implicitly condone the idea that less than fully functioning human behavior is not so much the result of the social environment, but of a deficit in the person themselves (an absence of a strength), and thus putting the responsibility on the person to manage or cope better in adverse circumstances.

Positive psychologists might not always be the people behind such interventions, but it is the technology and tools of positive psychology which are used when stressed and overworked employees are forced by their managers to attend well-being sessions, or school children are given mindfulness classes to cope with the mental health concerns. The gap that exists between research and practice might blind some to how their research is understood and used, and how ultimately its implementation may condone ideas about deficit and dysfunction within the person. In sum, if we reflect on the demise of humanistic psychology relative to the success of positive psychology, we might wonder if the latter's rise was at least in part because it fits well with the demands of conservative ideologies and the need for many organizations and institutions to control and coerce people to behave in particular ways, which presupposes an ideological position that runs counter to a growth model.

### The Subtle Use of Power in Positive Psychology

One of the ways in which psychology has power over people is through its adoption of the medical model. Humanistic psychology has long challenged the traditionally accepted parameters in psychology, including the model of a practitioner taken from medicine (Bugental, 1963). Rogers' (1959) approach succeeded in doing this because of how he theorized the nature of psychological problems as having a unitary cause in incongruence and he offered a form of therapy which was about the social environment; in these ways he moved beyond a separation of the negative and the positive into distinct fields of study, and the need for practitioners to take an expert stance over the person's inner experiences. In this way, the person-centered approach offers a different understanding of the power relations between practitioners and clients. Positive psychology promises to offer an alternative to the medicalisation of human experience (Maddux and Lopez, 2015), but yet it does so only in the most superficial of ways by not using the language of medicine but continuing to condone the essential elements of the medical model (see Joseph and Linley, 2006b).

The first way it does this is because the remit of positive psychology is often seen as a supplement to traditional psychology, which focuses on distress and dysfunction. In doing this it serves to condone the idea that there is a separation between the clinical and the more fully functioning aspects of human experience. The person-centered conceptualization is that while there is a universal human tendency toward actualization, this tendency becomes thwarted in non-optimal social environments, which create an incongruence between the tendency toward actualization and self-actualization. As such, it is usual for people to self-actualize in ways that are less than fully functioning. In this way, person-centered therapy effectively posits a unitary cause of distress, but varied expressions of that distress will arise according to the uniqueness of each individual's incongruence (see Sanders and Joseph, 2016).

A second way in which positive psychology continues to condone an illness ideology is through the notion that different interventions are needed for different states of positivity. It is a common assumption in clinical psychology that different interventions are needed for different clinical states, referred to as the specificity myth by person-centered psychologists (Bozarth and Motomasa, 2005). It is an assumption from clinical psychology that is applied to positive psychology that there are specific interventions for specific positive psychological states. But it is an assumption that runs counter to the person-centered proposal that there is a unitary cause of distress and growth, and thus a single form of intervention. Shlien (1989) wrote:

‘Client-centered therapy has only one treatment for all cases. That fact makes diagnosis entirely useless. If you have no specific treatment to relate to it, what possible purpose could there be to specific diagnosis? Nothing remains but the detrimental effects.' (p. 402).

The need for diagnosis, formulation, and all expressions of expertise over the person dissolve when it is the social environment that is the focus of intervention, not the person (Joseph, 2021). In this way, the positive psychologist may not be using diagnosis in the clinical sense, but if they are developing an intervention suitable for some people but not others, the same logic applies. As such, much of contemporary positive psychology remains underpinned by the medical model, but that fact is disguised by its language of strengths, virtues, and happiness. In sum, the assumption that different problems or people require different interventions leads the practitioner to take an expert stance, implying that they know what the client needs better than the client knows themselves, which presupposes an ideological position that runs counter to a growth model.

## Conclusions

As described above, reflection on positive psychology from the perspective of the person-centered approach leads to questions about the positionality of positive psychology, its politics, and its subtle promotion of power. The adoption of a growth model leads to a different way of addressing these same issues.

A growth model offers: (1) an alternative nomological net of variables for research, to do with the quality of relationships, growth promoting climates, and fully functioning personality dimensions, with (2) different implications for practice, to do with non-directive rather than directive interventions, and (3) significance in terms of real-world relationships between people, institutions, and society, as the aim is to work toward a social environment free from corruption, injustice, oppression, and poverty, and all other ways in which the growth of people is usurped and thwarted.

For researchers, this offers new challenges to understand whether and in what ways people will be intrinsically motivated to move in positive psychological directions when in optimal social environments, and how to define the optimal social environment, across different contexts and cultures. It alerts researchers that research findings in themselves do not indicate an approach to an intervention, and that there is a need to understand the relative merits of directive and non-directive approaches. It also helps us think about how our research is used by others and what other agendas our findings might be used to serve.

Such a shift in thinking would also have implications for what it means to be a positive psychologist. For example, in psychotherapy and clinical psychology, the practitioner must learn new ways of relating to people. Or in education, the educationalist must learn to trust in their students that they have the intrinsic need to learn and to develop. Adopting a person-centered approach to practice offers challenges to positive psychologists in terms of their own psychological development. Because the person-centered approach focuses on the relational climate that the practitioner fosters through their ability to be genuine, empathic, and congruent, the importance of the practitioner's own psychological development and emotional maturity cannot be understated.

Positive psychology is a broad discipline of study and practice. It isn't defined in terms of its approach. Positive psychologists take a variety of approaches to their work, including a person-centered approach, although it may not always be recognized as such.

While my own vision is for a more person-centered positive psychology, and that is the branch of positive psychology that I identify with most strongly, it might be said by some that it is a strength of positive psychology that it has no single paradigmatic positionality on human nature, as that allows for great flexibility in exploration, crossing between ideas and assumptions about human nature traditionally associated with the psychoanalytical, behavioral, and humanistic. As a discipline I would agree that positive psychology need not take any single paradigmatic stance. But that is not the same as it being neutral, as each instance of research or practice does have a stance, whether it is made explicit or not. Unless each researcher and practitioner acknowledges their own positionality, and describes how their focus of interest, measures chosen, and so on, arises from their point of view, what otherwise appears like a coherent and building body of knowledge is actually founded on a tangle of different assumptions. What could be a strength is a weakness. It is a weakness when positionality in research and practice is implicit and unacknowledged, as if it were not true that all research and practice comes from a position, as it allows for the fact that all research and practice is ultimately ideological to go unnoticed.

Seen like this, positive psychology provides a smorgasbord of methods, lacking in any single underpinning ontological approach. In this respect, positive psychology is not person-centered, but person-centered psychology can be thought of as a specific approach to positive psychology. Recognizing it as such places a much-needed new stake firmly in the ground to draw attention to, and create a tension with, whatever the other implicitly accepted ontological stances of mainstream positive psychology are, and which often imply that people's intrinsic motivation cannot be relied upon.

In these ways, I believe that positive psychology can learn from the person-centered position, to realize the often dark and destructive images of humanity that actually lie at the core of much contemporary positive psychology, disguised by its language of positivity. Despite the similarity in stated goals there can be gulf between humanistic and positive psychology. To close the gap, perhaps it may be helpful for positive psychologists to revisit Bugental's (1964) five basic principles of humanistic psychology and make them their own. Moving forward with a new research agenda, positive psychologists must become more explicit about their own positionality, to be clear what theoretical assumptions underpin their choice to focus either on the person or the social environment. Respecting the humanistic image of the human being and, consequently, considering and influencing people's social environment to facilitate personal growth would promote cross-fertilization between positive psychology and the person-centered approach instead of widening their gap. It would be useful for positive psychologists to be open regarding their image of humanity, thus offering positive psychology as an umbrella for interventions from different theoretical foundations and making that explicit would seem a step forward for positive psychology and a door-opener to include the person-centered approach.

In summary, while the move toward studying the good life is surely to be welcomed, in taking up the baton from humanistic psychology, positive psychologists left behind what I believe is the most vital part of the humanistic approach—its view of human nature. Whereas, humanistic psychology and specifically the person-centered approach provided an alternative growth paradigm to the behavioral and psychoanalytical schools that had come before, positive psychology as a whole takes no single paradigmatic stance. This might be seen as a strength for a discipline, but it is misleading to think that this means that each instance of research or practice is not based in a paradigm. Positive psychology may use the language of positivity, yet implicitly condone ideas about deficit and dysfunction within the person, and talk about growth, yet promote practices that quietly curtail freedom and self-direction. In this way, positive psychology may yet learn from humanistic psychology that our ideas about how to treat people are always based in our visions of human nature.

## Data Availability Statement

The original contributions generated for the study are included in the article/supplementary material, further inquiries can be directed to the corresponding author/s.

## Author Contributions

The author confirms being the sole contributor of this work and has approved it for publication.

## Conflict of Interest

The author declares that the research was conducted in the absence of any commercial or financial relationships that could be construed as a potential conflict of interest.

## Publisher's Note

All claims expressed in this article are solely those of the authors and do not necessarily represent those of their affiliated organizations, or those of the publisher, the editors and the reviewers. Any product that may be evaluated in this article, or claim that may be made by its manufacturer, is not guaranteed or endorsed by the publisher.

## Footnotes

^1^It is beyond the scope of this paper to go into the detail, but it is worth noting that this view of human nature was also taken up and developed subsequently by Ryan and Deci (2000) in their self-determination theory, which, by and large, offers theory and evidence consistent with and supportive of Rogers' theory (see Sheldon and Kasser, 2001; Patterson and Joseph, 2007; Joseph and Murphy, 2013b; Sheldon, 2013).
